# Supplementary figures and images for: Biological boundary conditions regulate the internalization of Aspergillus fumigatus conidia by alveolar cells
Source: Front Cell Infect Microbiol. 2025 Feb 24;15:1515779. doi: 10.3389/fcimb.2025.1515779 (PMC11891256; doi:10.3389/fcimb.2025.1515779)

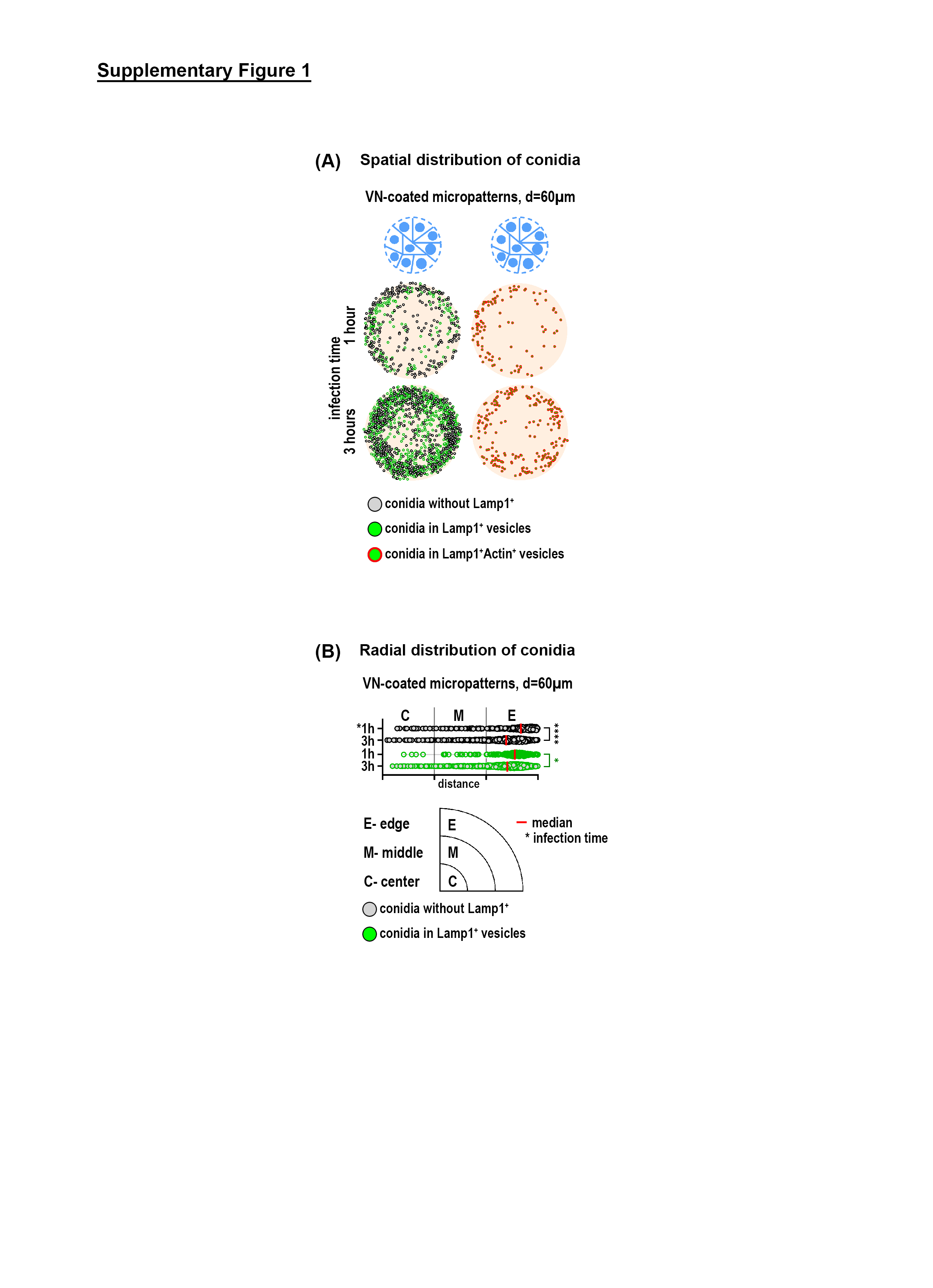

Supplement: Supplementary Figure 1 — Distribution and internalization of A. fumigatus conidia in A549 cells constrained on vitronectin-coated micropatterns. (A) Conidia spatial distribution map of 50 overlaid micropatterns displaying conidia without Lamp1 (grey spheres), conidia in Lamp1+ vesicles (green spheres) and conidia in the subpopulation of Lamp1+Actin+ vesicles (green spheres in red circles). (B) The radial distribution of conidia defined as the distance between the micropattern center and the center of a single conidia. These distances were grouped into three segments (center, middle and edge of pattern). Depicted are conidia without Lamp1 (grey spheres) and conidia in Lamp1+ vesicles (green spheres). [file Image1.tif]
